# Pancreatic PEComa: Case Report of an Extremely Rare Tumor

**DOI:** 10.34172/aim.34740

**Published:** 2025-12-01

**Authors:** Dmitry Zinovkin, Denis A. Davydov, Pavel G. Kisialeu, Diana A. Kolbik, Sergey L. Achinovich, Anna S. Portyanko, Md Zahidul Islam Pranjol

**Affiliations:** ^1^Department of Pathology, Gomel State Medical University, Gomel, Belarus; ^2^National Molecular Genetics Laboratory of Cancer Research, N.N.Alexandrov National Cancer Center of Belarus, Minsk, Belarus; ^3^Department of Pathology, Gomel Regional Oncological Clinics, Gomel, Belarus; ^4^School of Life Sciences, University of Sussex, Brighton, UK

**Keywords:** Diagnostic challenges, Immunohistochemistry, Pancreatic PEComa, Rare mesenchymal tumor, Surgical resection

## Abstract

Pancreatic perivascular epithelioid cell tumors (PEComas) are rare mesenchymal neoplasms with only a few reported cases. Their non-specific clinical presentations and imaging features often lead to misdiagnosis. We report a case of a 63-year-old female with intermittent left upper quadrant pain. Imaging revealed a hypervascular mass in the pancreatic tail, initially suspected to be a neuroendocrine tumor. The patient underwent distal pancreatectomy with splenectomy. Histopathological examination showed that the tumor consisted of epithelioid and spindle cells with clear cytoplasm, a rich vascular network and low mitotic activity. Immunohistochemically, the tumor cells were positive for HMB-45, Melan-A, and smooth muscle actin, confirming the diagnosis of pancreatic PEComa. The postoperative course was uneventful. Given the uncertain malignant potential of PEComas, complete surgical excision is the preferred treatment option, with long-term follow-up recommended. This case highlights the diagnostic challenges of pancreatic PEComas and underscores the role of histopathology and immunohistochemistry in their accurate identification and management.

## Introduction

 Perivascular epithelioid cell tumors (PEComas) are rare mesenchymal neoplasms characterized by the presence of perivascular epithelioid cells that co-express melanocytic and smooth muscle markers. While these tumors have been reported in various anatomical locations, including the kidneys, lungs, liver, and uterus, their occurrence in the pancreas is extremely rare.^1^ The first documented case of a pancreatic PEComa was reported by Zamboni et al in 1996, and since then, only about 30 cases have been described.^2^

 Pancreatic PEComas present with diverse clinical manifestations, ranging from incidental detection to symptoms related to mass effect, such as abdominal pain, weight loss, or obstructive complications.^3^ Due to their rarity and non-specific radiological features, pancreatic PEComas are often challenging to diagnose preoperatively. Histopathological and immunohistochemical evaluations are essential for confirmation, as tumors typically express melanocytic markers such as HMB-45 and Melan-A, in addition to smooth muscle markers like α-smooth muscle actin.^3^

 The biological behavior of pancreatic PEComas remains uncertain; some cases exhibit benign features, while others demonstrate aggressive growth, local invasion, or metastatic potential.^4^ Given their unpredictable nature, surgical resection is generally considered as the primary treatment option, especially for tumors with worrisome histopathological characteristics.^5^

 In this report, we present a case of pancreatic PEComa, detailing its clinical presentation, diagnostic findings, histopathological features, and treatment approach. This case contributes to the existing literature and emphasizes the importance of accurate diagnosis and appropriate management of these rare pancreatic neoplasms.

## Case Report

 A 63-year-old female patient presented with a two-year history of intermittent pain in the left upper quadrant. She had no significant past medical or surgical history. On admission, her weight was 94 kg, height 164 cm, and blood pressure 130/80 mmHg. Electrocardiography revealed sinus bradycardia (58 bpm), left axis deviation, and first-degree atrioventricular block. Laboratory tests showed mild anemia (hemoglobin 109 g/L), thrombocytosis (510 × 10⁹/L), and an elevated erythrocyte sedimentation rate (56 mm/h). Biochemical parameters were largely within normal limits, except for hyperglycemia (12.0 mmol/L) and a slightly elevated alkaline phosphatase level (136.1 U/L).

**Figure 1:**
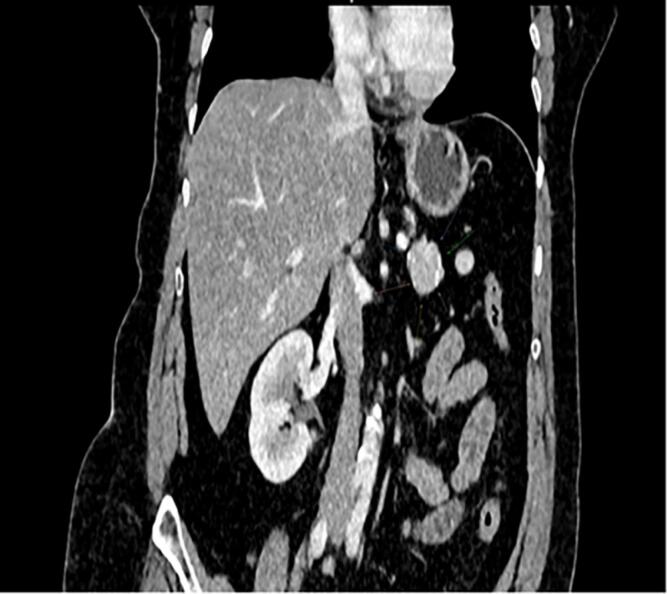
Well-circumscribed solitary tumor of the pancreas. Contrast-enchanced CT scan, sagittal plane

 Abdominal contrast-enhanced multi-slice computed tomography revealed a hypervascular mass measuring 30 × 21 × 25 mm in the tail of the pancreas (Figure 1), suspected to be neuroendocrine neoplasm. The lesion demonstrated early arterial enhancement with persistent contrast uptake in the venous phase, without pancreatic duct dilatation or invasion of adjacent structures. There were no radiologic signs of regional lymphadenopathy or distant metastases. Given the well-circumscribed nature of the mass and its location in the pancreatic tail, the surgical team opted for upfront resection instead of preoperative endoscopic ultrasound-guided fine-needle aspiration (EUS-FNA), to both avoid procedure-related risks and obtain a complete specimen for histopathological and immunohistochemical analysis. The patient underwent a distal subtotal pancreatectomy with splenectomy.

 Macroscopic examination of the resected specimen showed a well-circumscribed, solid, lobulated tumor in the pancreatic tail, measuring 30 × 21 × 25 mm, surrounded by a fibrous capsule. The lesion appeared pale brown in color. The spleen, measuring 95 × 45 × 55 mm and weighing 120 g, had an intact capsule except for a minor tear at the hilum.

**Figure 2:**
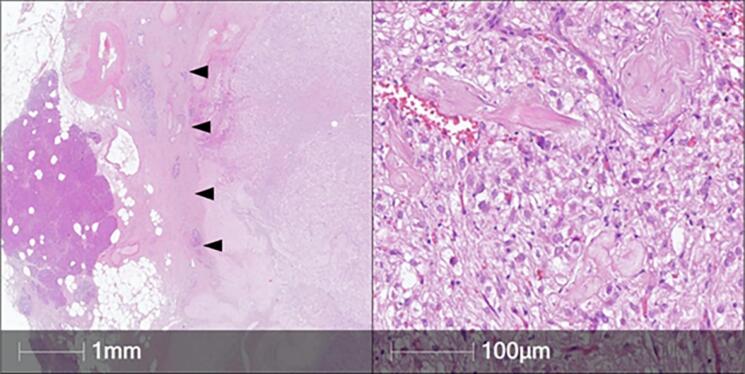
Histology of the Pancreatic Tumor. Hematoxylin & eosin, original magnification × 5 (left), × 200 (right). Left: the tumor is separated from pancreatic parenchyma by a broad fibrous capsule (arrowheads). Right: large tumor cells with clear and granular cytoplasm had a relatively bland appearance

 Histopathologically, the tumor had a thick fibrous capsule at the periphery, separating the lesion from the adjacent pancreatic tissue. The tumor consisted of large, predominantly epithelioid cells with clear and granular cytoplasm, round nuclei without prominent nucleoli. Occasional multinucleated cells were found, as well as minor areas of spindle-cell architecture. The mitotic rate was low, and necrosis was absent (Figure 2).

**Figure 3:**
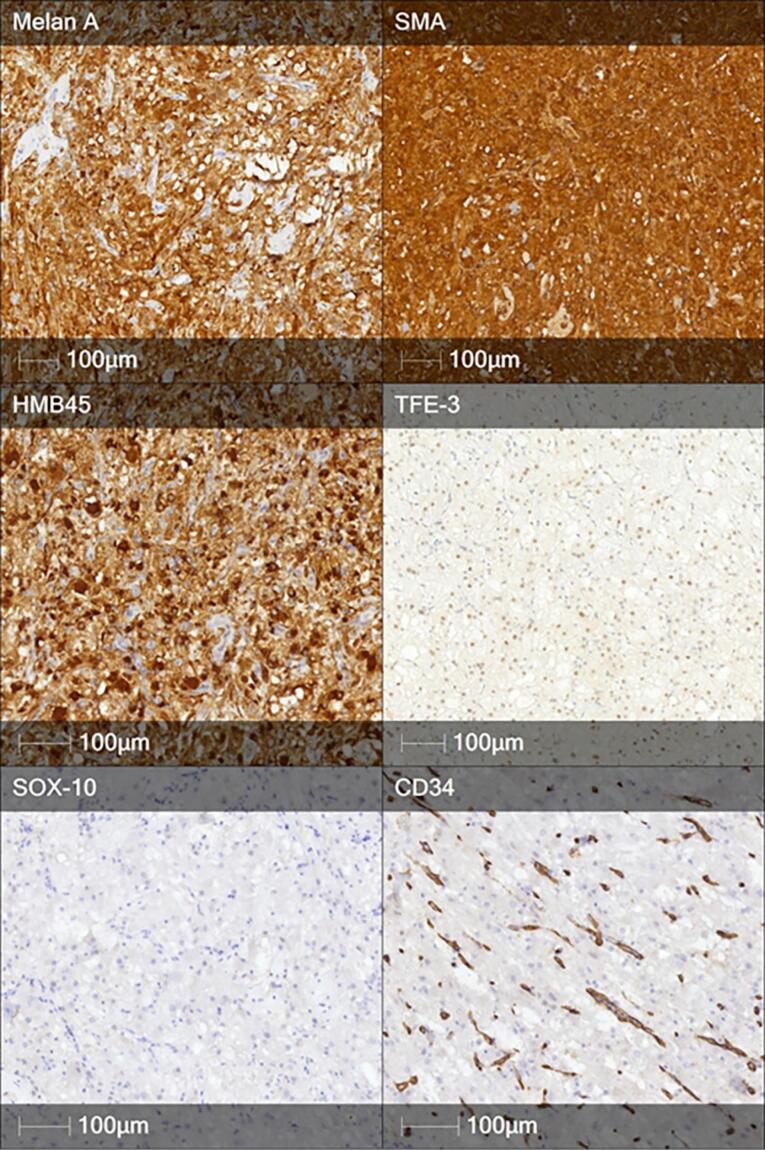
Immunophenotype of the Tumor. Immunohistochemistry, chromogen – DAB, counterstaining – Mayer’s hematoxylin, original magnification × 100

 Immunohistochemistry showed positivity of tumor cells for Melan A, SMA, HMB-45, TFE-3. Tumor cells were negative for SOX-10, CD34, Pan-cytokeratin, S100, CD56, Desmin (Figure 3). Proliferation index (Ki-67) measured less than 1%. These findings confirmed the diagnosis of pancreatic PEComa.

 The postoperative course was uneventful, with no signs of complications. Drainage fluid analysis showed normal amylase levels (56.16 U/L). The patient was discharged in good condition after 7 days.

## Discussion

 Pancreatic PEComas are exceedingly rare mesenchymal neoplasms, with only a limited number of cases reported in the literature.^6^

 Their pathogenesis, clinical behavior, and optimal management remain incompletely understood. This case report contributes to the growing body of literature on pancreatic PEComas, emphasizing the diagnostic challenges, the role of histopathology and immunohistochemistry, and the necessity for long-term surveillance. Pancreatic PEComas often present as incidental findings or with non-specific symptoms such as vague abdominal discomfort, weight loss, or gastrointestinal disturbances. In our case, the patient experienced intermittent left upper quadrant pain over two years, an insidious presentation that aligns with prior reports.^6^ Given their rarity and the absence of pathognomonic radiologic features, pancreatic PEComas are frequently misdiagnosed preoperatively. Contrast-enhanced imaging typically reveals a hypervascular lesion, often leading to an initial impression of a neuroendocrine tumor or another hypervascular pancreatic neoplasm.^7^ Although EUS-FNA has been reported as a valuable minimally invasive method for preoperative tissue diagnosis of pancreatic masses, its role in PEComas is limited due to the rarity of the lesion and the difficulty in obtaining adequate material for definitive immunohistochemical evaluation. In several published cases, cytological smears were inconclusive or misleading, and only the cell block preparation with extended immunohistochemical panel raised suspicion of PEComa.^5,8,9^ In our patient, EUS-FNA was not performed preoperatively because the lesion was small, hypervascular, and surgically accessible, and because intraoperative resection would provide sufficient tissue for definitive diagnosis without the potential risks of needle tract seeding or hemorrhage.

 However, in many cases, including ours, a definitive diagnosis is only established postoperatively through histopathological and immunohistochemical evaluation.^6^ This highlights the ongoing challenge of distinguishing PEComas from other pancreatic tumors based on imaging alone.

 Histopathological examination remains the gold standard for diagnosing pancreatic PEComa. The tumor in our case exhibited the classical features described in the literature: a proliferation of epithelioid and spindle cells with abundant clear and granular cytoplasm, a rich vascular network and low mitotic activity. These findings are consistent with prior reports, reinforcing the characteristic histological profile of PEComas. Immunohistochemically, PEComas demonstrate dual melanocytic and smooth muscle differentiation. Specifically, the combination of strong HMB-45, Melan-A, and SMA positivity, together with negativity for SOX-10, CD34, pan-cytokeratin, and S100, effectively rules out most histological mimickers.^10^ For example, clear cell carcinoma of the pancreas or metastatic renal cell carcinoma will show cytokeratin expression, Gastrointestinal stromal tumors are typically possitive for CD117 and DOG1 positive, melanomas are S100 and SOX-10 positive, and leiomyosarcomas express SMA and desmin but lack melanocytic markers. This underlines the diagnostic value of a broad immunohistochemical panel in distinguishing PEComas from other clear cell and spindle cell pancreatic neoplasms.^11^

 Surgical resection remains the mainstay of treatment for pancreatic PEComas, particularly in cases where the malignant potential is uncertain. In our case, a distal subtotal pancreatectomy with splenectomy was performed, consistent with standard surgical approaches for pancreatic tail tumors.^12^ The patient’s postoperative course was uneventful, and she was discharged in stable condition, mirroring outcomes in other reported cases where complete resection resulted in a favorable short-term prognosis. Despite their often indolent behavior, the biological potential of pancreatic PEComas remains a subject of debate. While many cases exhibit benign behavior, others demonstrate aggressive features, including local recurrence and distant metastasis.^13^ The risk stratification criteria proposed by Folpe et al suggest that PEComas with a size larger than 5 cm, high mitotic rate, necrosis, vascular invasion, or infiltrative growth may have a higher malignant potential.^14^ In our case, the tumor measured 3 cm, had low mitotic activity, and lacked necrosis or vascular invasion, suggesting a low risk of malignancy. However, given the unpredictable nature of PEComas, long-term follow-up is warranted.

## Conclusion

 Pancreatic PEComa is a rare and diagnostically challenging entity. Its non-specific clinical presentation and imaging characteristics require a high index of suspicion, with definitive diagnosis depend on histopathological and immunohistochemical confirmation. Surgical resection remains the primary treatment for pancreatic PEComas, offering favorable outcomes in most cases. However, due to the unpredictable biological behavior of these tumors, long-term surveillance is essential. As more cases are documented, a clearer understanding of their clinical course, molecular characteristics, and optimal management strategies will emerge. This progress will pave the way for standardized diagnostic and therapeutic guidelines.
